# Collaboration of health and education sectors drives equity for children with complex disabilities in China

**DOI:** 10.3389/fpubh.2023.1292491

**Published:** 2023-11-07

**Authors:** Lisa Jacobs, Deborah Gleason, Daniela Gissara, Nathan Congdon, George Smith, Peter Xu

**Affiliations:** ^1^Perkins School for the Blind, Watertown, MA, United States; ^2^Queen’s University Belfast, Belfast, Northern Ireland, United Kingdom; ^3^Zhongshan Ophthalmic Center, Guangzhou, China; ^4^Orbis International, New York, NY, United States

**Keywords:** children with disabilities, complex disabilities, visual impairment and blindness, family-centered care, low- and middle-income countries, healthcare and education, inclusion, home visiting

## Abstract

Medical professionals often find it challenging to assess children having both complex disabilities and visual impairment, which may lead to excluding such children from educational programs and limiting their full participation in family and community activities. Identification and assessment of these children are essential to close this exclusion gap. A five-year project in Shanxi province, China, provided comprehensive training to eye health providers and educators as they learned to assess, identify, refer and serve children with visual impairments, both with and without complex disabilities. A team of teachers, vision and general healthcare providers worked to assess the vision of these children at schools, residential settings, and in homes throughout Shanxi. The project led to deep collaboration between Shanxi’s health and education sectors, and established replicable precedents for policy and system changes toward the inclusion of children with complex disabilities and visual impairment.

## Introduction

Schools’ unique ability to reach all levels of society positions them to play a crucial role in children’s healthcare. They are efficient platforms for conducting health assessments benefiting a broad range of children, and for connecting families to additional services and care. However, children with complex disabilities are often left out of this process, leading to their exclusion from services, an issue that is especially persistent in low and middle income communities across the globe (1).

Orbis International, Perkins School for the Blind and their Chinese partners confronted this issue in Shanxi province as part of Standard Chartered Bank’s *Seeing is Believing* program, a global initiative to address preventable blindness and improve education and rehabilitation services for children. The five-year project’s goal was to identify children with visual impairment and complex disabilities in rural counties surrounding four cities in Shanxi province: Taiyuan, Datong, Jincheng and Jinzhong, and to help close the exclusion gap by connecting the children and their families to vision care and education services.

Progress toward achieving the United Nations Sustainable Development Goals was also an important project throughline. The specific goals targeted were: #3, ensure healthy lives and promote well-being for all at all ages; #4, ensure inclusive and equitable quality education and promote lifelong learning opportunities for all; and #17, strengthen the means of implementation and revitalize the Global Partnership for Sustainable Development. Key collaborators in addition to Orbis and Perkins included the Shanxi Provincial Bureau of Education, Shanxi Provincial Eye Hospital, Zhongshan Ophthalmic Center in Guangzhou, Brien Holden Vision Institute, and Helen Keller International.

This article sheds light on the partnerships among project collaborators from the health and education sectors that ultimately led to widespread project success. The commitment of Chinese doctors, teachers, and government officials was a key factor in sustaining this collaborative approach to drive equity for children with complex disabilities in China.

Three key aspects of the project are explored here: identifying children with visual impairment and complex disabilities in low resource settings, establishing a connection between healthcare and education, and the importance of taking a family-centered approach to care while fostering collaborative models to support these children. Each of these areas presents its own set of rewards and challenges and the lessons learned are also shared throughout this article.

## Key project components

### Identifying children with visual impairment and complex disabilities in low resource settings

The identification of children with visual impairment was a primary project goal. School-based screenings and traditional standardized testing tools worked well to identify these children, because they were already attending school, and the testing tools were appropriate for their developmental level. As the project progressed, however, it became clear that these strategies were ineffective for an entire population that was in great need of education, medical care and other services: children with visual impairment and additional complex disabilities.

In the project’s low- and middle-resource settings in Shanxi province, most children with complex disabilities were not attending school. Consequently, these children were excluded from school-based screenings, and thus remained unidentified by the systems that could provide support to them and their families (2). They were essentially unknown and invisible to providers of educational services, critical medical eye care, social services, and numerous other supports.

Another significant barrier to identification of children with complex disabilities that became apparent during the project is the failure of traditional testing methods to identify these children. The small number of children with complex disabilities who were attending school, and therefore had the opportunity to be screened, were still not being identified accurately due to the types of tests being used. Standard vision testing tools like the Snellen Chart use letters of varying sizes to measure visual acuity. It relies on a child’s use of language and recognition of letters, as the child must indicate verbally what s/he can and cannot see during an assessment. Many children with complex disabilities did not have the developmental and language skills needed to complete this type of assessment. Children who could not respond to a vision assessment in the typical way were then labeled as “untestable” by doctors and vision screeners, which excluded them from accessing services and vision care.

These two key issues, children with complex disabilities not being enrolled in school, and use of conventional screening methods that were ineffective, resulted in a large number of children in Shanxi province being excluded from services, education and care. Discovery of these issues led to a new direction for the project. To ensure inclusivity in assessments, Perkins, Orbis and local partners took a proactive and comprehensive approach that linked the healthcare and education sectors. This initiative focused on training and equipping medical professionals and educators with the skills to assess, identify, refer and serve children with complex disabilities.

### Linking healthcare and education for children with complex disabilities

“Before training, I thought low vision was just a visual impairment. I didn't know that I could help children to fully use their vision. I not only learned to assess functional vision for children with low vision, I also can be patient and…serve every single child with disabilities, and help them to have fair eye care.”—Clinician at Yangcheng Eye Hospital

Children with visual impairments and complex disabilities are a diverse population with unique needs (3). Accurate assessment of these children to identify their level of visual impairment and then connect them with appropriate education and health services requires methods that are tailored to each child, and must be led by a team of professionals (4). In order to build this team, it was crucial for the project to foster a partnership between Shanxi’s health and education sectors.

The partnership involved both eye health providers and educators, and the approach focused on training professionals and on the development of essential facilities. Comprehensive training led by Perkins and Orbis provided professionals with the skills and tools needed to assess, identify, and support children with visual impairment and complex disabilities. Village doctors, key informants including community leaders and social workers, and Chinese Disabled Persons’ Federation workers also participated in trainings, providing a critical link for reaching children who were isolated at home with no access to services. Partners were also equipped with facilities, including low vision clinics, toy libraries, and a Low Vision Educational Resource Center. These initiatives proved invaluable, providing professionals with the necessary resources for accurate and appropriate vision assessments.

The project’s comprehensive and collaborative approach to training prompted transformative shifts in practitioners’ mindset toward children with complex disabilities. Eye doctors who had previously dismissed these children as “untestable” embraced the new assessment methodologies and put them to use. They were trained in clinical testing tools and strategies appropriate for children with complex disabilities, such as LEA Symbols for assessing children with limited verbal language, and Preferential Looking Test cards, which are useful for children who are unable to identify symbols or pictures of any kind.

Teachers were trained in Functional Vision Assessment and Learning Media Assessment, so that they could learn to observe how children use their vision at home, school and in natural environments. Teachers then could share those observations with families and eye doctors, in a truly collaborative partnership. Teachers also learned how to include vision accommodations in their classrooms and curricula and how to guide families in their use at home.

All practitioners learned how to work together and with parents in vision assessments. They developed new ways of looking at children with complex disabilities, a new approach to teamwork, and new skills around assessments and home visits. This led to substantial improvements in the identification of children with visual impairments and complex disabilities, enhancing access to medical care, tailored support, and educational opportunities.

### Family-centered care and collaboration models of care for children with complex disabilities

There was a child with multiple disabilities who lived in Shanxi province. The family was disconnected from local resources and did not know how to support their child. The project team visited the family at home and conducted a vision assessment, then taught the parents communication and teaching strategies, and modified their home to better meet the child’s needs. They also connected the family to a local school, where the child was enrolled within a week.

Because conducting assessments through schools was ineffective for identifying children with visual impairment and complex disabilities, it was important for the project to establish alternative ways to reach these children. Backed by recent research that robustly supports home visits as an effective method for delivering child development services and family support (5, 6), the project team began to implement this approach. The goal for home visits was to have a team of trained health and education professionals assess the child’s vision and also provide training and support to family members, connecting them with educational and medical professionals to coordinate services in preparation for the child’s entry into school.

Collaboration with village doctors and local key informants was a crucial part of finding the children who were unenrolled in school and in need of services. These local collaborators were trained in recognition and referral of children potentially at risk for visual impairment and complex disabilities. Once children at risk were identified, a team of local eye doctors, teachers, and project staff traveled together from village to village, visiting the children and families in their homes. The team assessed each child’s functional vision, spent time discussing the child’s diagnosis with parents and other caregivers, and taught them how to make their homes into accessible learning environments.

This new collaboration among teams of health and education professionals enabled them to successfully provide family-centered care, intervention and education for children with complex disabilities who were previously disconnected from services. Access to inclusive, equitable education in the region was greatly improved, and the project also resulted in widespread changes in perception among doctors, teachers, and families about what is possible for these children.

## Lessons learned

The project led to numerous successful practices in the field of vision care for children with and without complex disabilities that have implications for both future practice and public policy. Three key areas rose to the top as important lessons learned that could be replicated on a broad scale with the appropriate public policies in place. They are: improving collaboration between medical and education sectors; meeting children’s needs through home visits; and the importance of leadership commitment. Each of these are described below, with policy recommendations discussed in the final section.

### Enhancing collaboration between medical and education sectors

"The information sharing with teachers helped us to understand these patients’ lives, and learning status, and barriers…we could then provide teachers more meaningful advice.”—Shanxi Medical Professional

While enhancing the collaboration between medical and education sectors was not an originally intended outcome, it proved to be a transformative approach that benefitted not only children with complex disabilities, but all partners and families involved. The value of having doctors and teachers work together, rather than independently of each other, was evident in numerous ways. Eye doctors were empowered to make more accurate and meaningful diagnoses because they were able to integrate the teachers’ perspectives and observations about the child into their diagnostic process. Additionally, the doctors’ assessments and recommendations provided important information to schools about each child’s usable vision, which resulted in teachers creating and providing the adapted materials those children needed to be successful in the classroom.

The doctors and teachers in China welcomed the collaborative process, and displayed a resolute “can-do” attitude when it came to locating and teaching children with visual impairments and complex disabilities. Practitioner commitment evolved even further when professionals from Shanxi province Eye Hospital traveled to Perkins in Boston to observe health/education collaboration and reflected on what this model could mean for their country.

Ultimately, the cross-sector training of over 40,000 professionals was instrumental in enhancing their capabilities, and led to 1.32 million children being screened for visual impairments, and identification of 1,363 children with visual impairment and complex disabilities (7).

Project success was also evident when eye specialists and teachers ventured into the field, traveling together to various rural Shanxi villages to find and identify the children who had been left behind. The practitioners involved were eager for more hands-on experience, as it reinforced their new belief in the power of collaboration and further strengthened their dedication to this transformative model. The two sectors worked cohesively to ensure that every child had equal opportunity for identification, assessment and support.

### Meeting children where they are: visiting homes

Building on the collaborative working relationships established between doctors and teachers, the project also confirmed how successful home visitation screenings can be in meeting the needs of children with complex disabilities. When the project shifted tactics to focus on home visits in order to reach children who were not attending school, over 400 children were identified in homes across the region (7).

Home visits also offered a way to build strong relationships between professionals and families, which laid the groundwork for ongoing support. Practitioners worked closely with parents and caregivers, adapting teaching strategies and home environments to create supportive learning spaces. This approach addressed the child’s immediate educational needs while also nurturing the family’s sense of agency and belonging within the community.

To bolster the success of home visits, the project invested in structured training and on-the-job mentoring for teachers. Perkins and local partners developed a home visit manual – available in both English and Mandarin – that played a crucial role in guiding teachers’ efforts (8). It provided guidance on both the philosophical and procedural aspects of home visits, teaching strategies, and instruction on preparing a child for school. This ultimately empowered teachers to conduct their own home visits, expanding the initiative’s reach and impact.

### Leadership commitment

Another key lesson from the project is the undeniable importance of leadership commitment to project outcomes. Key individuals from hospitals and government agencies emerged as passionate advocates for the welfare of children with complex disabilities and visual impairment (7). Their dedication and support were pivotal in driving the project’s success and ensuring its long-term impact.

Partnerships with the Ministry of Education, Ministry of Health, Chinese Disabled People’s Federation, the Department of Social Welfare, local hospitals, and schools were also essential to the project’s success. These organizations provided resources, expertise, and connections to key informants and village doctors, who played a critical role. This broad network of collaborators enabled the reach of a wide audience and supported the implementation of a comprehensive approach in caring for children with complex disabilities. As the models of collaboration between the education and medical sectors continue to evolve and expand their impact, the importance of leadership commitment remains at the forefront.

## Recommendations and conclusion

Findings from the project in Shanxi have broad implications for policy makers, with the potential to impact health and education systems across China and perhaps around the world. In 2014, China put a national policy in place requiring home visits for children not attending school. This policy was not implemented consistently across the country, however, and a formal recommendation and support from the Shanxi Provincial Bureau of Education in 2017 was key in ensuring that home visits could take place in this region.

The authors recommend that local formal recommendations for implementing this policy be adopted across China’s other provinces as well, as an important engine for inclusion of children with vision impairment and complex disabilities. Additional aspects of the Shanxi project that could be replicated in other regions to ensure that the national policy requiring home visits for children is appropriately implemented include:

1. Training for teachers in how to effectively provide educational services for children with complex disabilities in home-based settings.
2. Teams of professionals from both health and education sectors work collaboratively to provide services and home visits for these children.

The project was also successful in changing the insurance coverage policy in Shanxi regarding surgical correction of strabismus, a highly prevalent condition in children with vision impairment and complex disabilities. This condition was previously considered cosmetic, and therefore surgical correction was not covered under China’s rural health insurance system. The Shanxi project showed the association between children’s vision and their mental health (9, 10), which led to reclassification of strabismus surgery as medically necessary. The authors feel this reclassification should be replicated in all of China’s provinces.

While policy changes are an important step toward building inclusive health and education systems, in order for new policies to be implemented effectively, practitioners must also receive ongoing training to understand the unique needs of children with complex disabilities and visual impairment.

Assessment and identification of children with complex disabilities and visual impairments are key to connecting these children and their families to the education, health, and services they need to flourish. Through ongoing training and support, eye doctors and teachers in low and middle resource settings in Shanxi province learned to use an expanded range of assessment tools and strategies that better identify children with complex disabilities. This resulted in enhanced collaboration between the medical and education sectors, effective assessment through home visits to identify children who were not attending school, and strong commitment of local leaders to continue supporting this population of children for the long term.

Appropriate assessment and identification of children with complex disabilities and visual impairment, paired with connecting children and their families with educational services and trained teachers, should be a priority in low resource settings around the world. Inclusion in healthcare, education and support services are crucial for allowing these children to develop to their full potential.

## Data availability statement

The original contributions presented in the study are included in the article/supplementary material, further inquiries can be directed to the corresponding author.

## Ethics statement

Ethical review and approval were not required for this manuscript, in accordance with local legislation and institutional requirements in China. No original data of any kind was collected or presented. Written informed consent from the program evaluation key informants was not required to participate in this study in accordance with Chinese national legislation and the institutional requirements of Orbis International and our China-based partners.

## Author contributions

LJ: Writing – original draft, Writing – review & editing. DGl: Writing – original draft, Writing – review & editing. DGi: Writing – original draft, Writing – review & editing. NC: Writing – review & editing. GS: Writing – review & editing. PX: Writing – review & editing.
